# Development of novel nanocomposite adsorbent based on potassium nickel hexacyanoferrate-loaded polypropylene fabric

**DOI:** 10.1186/1556-276X-9-180

**Published:** 2014-04-13

**Authors:** Yuliia Bondar, Svetlana Kuzenko, Do-Hung Han, Hyun-Kug Cho

**Affiliations:** 1 Institute of Environmental Geochemistry, 34a Palladin ave, Kiev 03142, Ukraine; 2 School of Chemical Engineering and Technology Yeungnam University, 214-1 Dae-Dong, Gyeongsan 712-749, South Korea

**Keywords:** Nanocomposite adsorbent, Potassium nickel hexacyanoferrate(II), Nanoparticles, Polypropylene fabric, Radiation grafting, Selective adsorption, Cesium

## Abstract

A nanocomposite adsorbent based on potassium nickel hexacyanoferrate-loaded polypropylene fabric was synthesized for selective removal of Cs ions from contaminated waters by a two-stage synthesis: radiation-induced graft polymerization of acrylic acid monomer onto the nonwoven polypropylene fabric surface with subsequent *in situ* formation of potassium nickel hexacyanoferrate (KNiHCF) nanoparticles within the grafted chains. Data of scanning electron microscopy, X-ray diffraction, and Fourier transform infrared spectroscopy confirmed the formation of KNiHCF homogeneous phase on the fabric surface, which consisted of crystalline cubic-shaped nanoparticles (70 to 100 nm). The efficiency of the synthesized adsorbent for removal of cesium ions was evaluated under various experimental conditions. It has demonstrated a rapid adsorption process, high adsorption capacity over a wide pH range, and selectivity in Cs ion removal from model solutions with high concentration of sodium ions.

## Background

The selective removal of ^137^Cs ions from liquid radioactive waste and their quantitative determination in the environment have a great importance in recent years. Insoluble divalent transition metal hexacyanoferrates(II) are very effective inorganic adsorbents for cesium ions [1]. Because they possess a high selectivity for Cs binding in the presence of alkaline earth and alkali metal ions, several attempts have been made to use hexacyanoferrates (HCFs) for the treatment of liquid radioactive waste with high salt content [2,3].

However, HCFs are usually synthesized as fine or ultrafine grains which are difficult for practical applications due to their low mechanical stability and tendency to become colloidal in aqueous solution. In order to improve their mechanical properties, deposition of insoluble hexacyanoferrates on various solid supports has been suggested as a possible solution. Different composite adsorbents were fabricated by loading nanosized HCFs onto the surface or inside of pores of inert solid supports such as silica gels [4], zeolites [5], zirconium and titanium hydroxides [6], different organic ion exchangers [7,8], etc.

Fibrous natural and synthetic polymers with ion exchange groups are promising host solid support for the synthesis of composite adsorbent with nanosized HCFs. Such composite adsorbent is expected to combine the unique properties of nanoscaled HCF particles (high specific surface, high speed of chemical reactions, selectivity) and technological properties of fibrous polymer matrix (flexibility, chemical stability, high specific surface, low hydraulic permeability, and ease utilization in dynamic sorption regimes).

Among various hexacyanoferrates, one of the most promising cesium-selective reagents is potassium nickel hexacyanoferrate (KNiHFC), which displays high chemical resistance in acid and alkaline solutions, mechanical stability, and thermal stability [2,9]. Polypropylene (PP) fibers and nonwoven fabrics are very attractive support in preparing nanocomposite adsorbents because of the low cost, good mechanical strength, chemical and thermal resistance of the PP base, and highly developed specific surface of the fibrous structure.

In this study we propose a novel nanocomposite adsorbent based on a KNiHCF-loaded polypropylene fabric for Cs, which was prepared by the radiation-induced graft polymerization of acrylic acid monomer onto the surface of nonwoven polypropylene fabric, followed by *in situ* formation of KNiHCF nanoparticles within the grafted polyacrylic acid chains. The synthesized adsorbent was used for the removal of Cs ions from the model solutions in batch mode, and the influence of contact time, pH, and presence of sodium ions on the adsorption process was investigated.

## Methods

### Materials

Nonwoven material made of polypropylene fibers, available from Saehan Filter Co., Ltd. (Cheongju, South Korea), with an average thickness of 1 mm was used for the synthesis of the nanocomposite adsorbent. Analytical grade NiCl_2_ · 6H_2_O (Duksan Pure Chemicals Co., Ltd., Ansan-si, South Korea) and K_4_[Fe(CN)_6_] 3H_2_O (Sigma-Aldrich, St. Louis, MO, USA) were used to prepare experimental solutions, respectively.

Nonradioactive CsCl (Dae Jung Chemicals & Metals Co., Ltd., Shiheung City, South Korea) was used as a surrogate for ^137^Cs because of its identical chemical characteristics. All working solutions were prepared using deionized water; pH was adjusted with a suitable quantity of NaOH and HCl, monitored with a digital pH meter. All experiments were carried out at ambient temperature.

### Preparation of the KNiHCF-loaded polypropylene fabric

The composite material based on the nonwoven polypropylene fabric with chemically bound KNiHCF nanoparticles was synthesized through a two-stage experiment. At the first stage, the chemically inert polypropylene base was activated through the radiation-induced graft polymerization of acrylic acid monomer (AA) for the introduction of chemically active carboxyl groups onto the surface of PP fibers through covalent bonding between grafted polyacrylic acid (PAA) chains and PP base [10]. Grafted fabric samples with a medium value of AA grafting degree (120% to 170% and carboxyl group density of 6.0 to 7.5 mmol/g) were taken for the experimental work. The second stage consisted of Ni^2+^ ion loading onto PAA chains grafted to the PP fabric (PP-g-PAA) followed by the *in situ* formation and stabilization of KNiHCF nanoparticles within the grafted layer. For this purpose, the PP-g-PAA fabric was immersed in 0.1 M NiCl_2_ solution for 12 h. After filtration, washing with distilled water, and drying at ambient temperature, the resulting PP-g-PAA (Ni) fabric was added to 2.5% solution of potassium hexacyanoferrate(II) for 24 h under gentle mixing. Finally, the KNiHCF-loaded PP fabric was separated by filtration, washed with deionized water until clear rinsing solution, and dried at 60°C for 24 h.

### Characterization of the KNiHCF-loaded polypropylene fabric

The surface morphology of the original PP and KNiHCF-loaded PP fabrics was recorded by a Hitachi S-4100 field emission scanning electron microscope (SEM; Hitachi, Ltd., Tokyo, Japan) at an acceleration voltage of 15 keV. The elemental composition was performed by energy-dispersive X-ray spectroscopy (EDS). The studied samples were sputter-coated with a thin Pt layer prior to examination.

Fourier transform infrared (FT-IR) measurements were carried out using a Spectrum™ 100 FT-IR spectrometer (PerkinElmer, Waltham, MA, USA) with attenuated total reflectance (ATR) mode. Spectra were collected by cumulating 24 scans. X-ray diffraction studies were carried out on a DRON-3 diffractometer (Scientific Industrial Enterprise “Burevestnik”, St. Petersburg, Russia) using Cu-K_α_ radiation in the range 10° to 90° in 2*θ* at room temperature.

### Adsorption experiments

A cesium chlorite stock solution of 1,000 mg/l was diluted, as required, to obtain the desired concentration. The pH of the solution was adjusted by using dilute solutions of hydrochloric acid, or sodium hydroxide, depending on the requirement.

Adsorption experiments were carried out in batch mode under shaking by placing a dry nanocomposite fabric (0.1 g) in a series of polypropylene flasks with 20 ml of CsCl solution. Once the required time elapsed, the residual solution was filtered through a Whatman filter paper and analyzed for Cs concentration by the atomic absorption spectrophotometer model AA-8500 (Nippon Jarrell-Ash Co., Ltd., Kyoto, Japan).

The amount of Cs adsorbed by the synthesized nanocomposite adsorbent at time *t*, *Q*_*t*_ (mg/g), was calculated as follows:

$$Q_{t}=\frac{\left(C_{0}-C_{t}\right)\times V}{W}$$

where *C*_0_ and *C*_*t*_ are the initial concentration and concentration of Cs at time *t* (mg/l) in the experimental solution, *V* is the volume of the solution (l), and *W* is the weight of the adsorbent (g). At the equilibrium time, *Q*_*t*_ = *Q*_*e*_.

Adsorption efficiency *α* (%) at equilibrium was calculated as follows:

$$\alpha =\frac{C_{0}-C_{e}}{C_{i}}\times 100$$

where *C*_*e*_ is the cesium concentration at equilibrium.

All the experiments were performed in duplicate.

## Results and discussion

### Preparation of the KNiHCF-loaded polypropylene fabric

The nanocomposite adsorbent based on potassium nickel hexacyanoferrate-loaded polypropylene fabric was synthesized through a two-stage experiment: radiation-induced graft polymerization of acrylic acid monomer onto the nonwoven PP fabric followed by the *in situ* formation of KNiHCF nanoparticles within the grafted chains.

The first stage, which resulted in the synthesis of the PP fabric with grafted PAA chains with a wide spectrum of carboxylic group density, was examined previously [10]. The first stage is a very important one due to several reasons. First, it allows the activation of the chemically inert polypropylene base through covalent bonding between grafted PAA chains of nano/micro-sized length and the PP fibers' surface. As a result of the grafting process, the PP-g-PAA fabric surface became covered with cation exchange groups, which could be loaded with any metal ions.

Second, the grafted chains loaded with Ni^2+^ ions serve as precursors of KNiHCF nanoparticles. The formation of KNiHCF nanoparticles occur inside of the grafted chains, and thus, these nanoparticles become attached to the fibers' surface via both physical and chemical forces. Third, the characteristics of grafted chains (density, length, chemical nature of the functional group) make it possible to control the *in situ* formation of inorganic nanoparticles, namely their density of distribution, size, and morphology. Therefore, it is possible to consider the grafted chains as a ‘nanoreactor’ for the nanoparticles' formation. Furthermore, they stabilize and isolate the formed nanoparticles, thus preventing their aggregation. Thus, the grafted chains can open wide opportunities for the *in situ* synthesis of inorganic nanoparticles with tailored morphology and size.

The intent of the second stage consisted in the *in situ* formation of KNiHCF nanoparticles on the PP fibers' surface. The second stage involved Ni ion loading onto the grafted chains and subsequent reaction of PP-g-PAA (Ni) fibers with potassium hexacyanoferrate solution.

We believe that the close position of the charged carboxyl groups through the nano/micro-sized length of grafted PAA chains as well as the close position of the neighboring chains could have created the nucleation sites of Ni nanoclusters which, by subsequent reaction with potassium hexacyanoferrate, have led to the formation of KNiHCF nanoparticles within the grafted chains.

### Characterization of the KNiHCF-loaded polypropylene fabric

**Figure 1:**
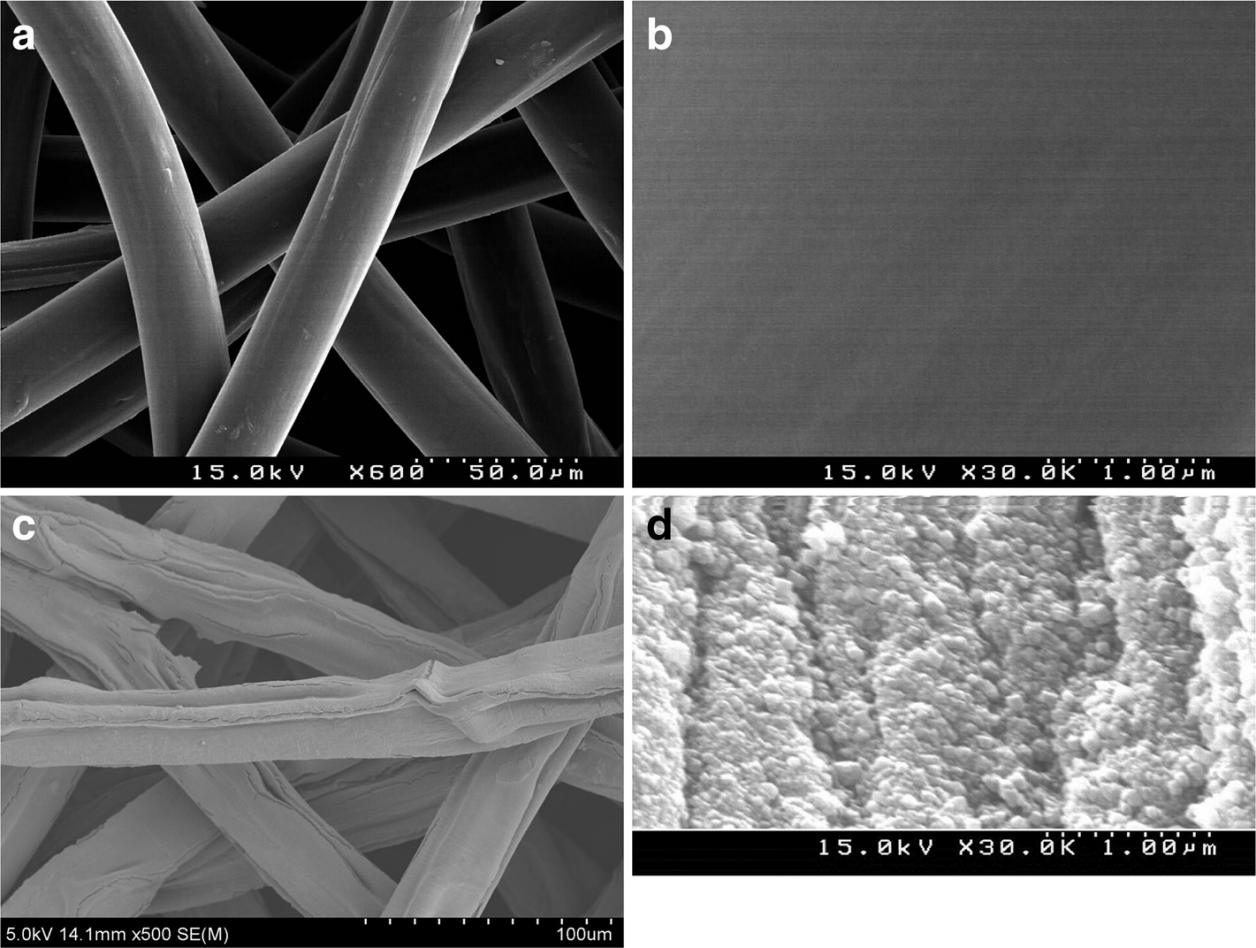
SEM images of PP-g-PAA fibers with 170% degree of acrylic acid grafting (a, b), and KNiHCF-loaded PP fibers (c, d).

Figure 1 shows the SEM images of the outer surface of the grafted PP fibers (degree of acrylic acid grafting is 170%) and the outer surface of the same PP fabric after loading of KNiHCF. The original and grafted PP fibers have a round shape, smooth surface, and cream color (Figure 1a,b). After loading of KNiHCF phase, these fibers changed their form and became greenish (Figure 1c). The SEM image at a higher magnification (Figure 1d) shows the surface morphology of the composite fibers with KNiHCF. One can see the fine single crystals (about 70 to 100 nm) of KNiHCF, which are cubic in shape. The KNiHCF nanocrystals fit one to another and form a compact texture on the fibers' surface.

**Figure 2:**
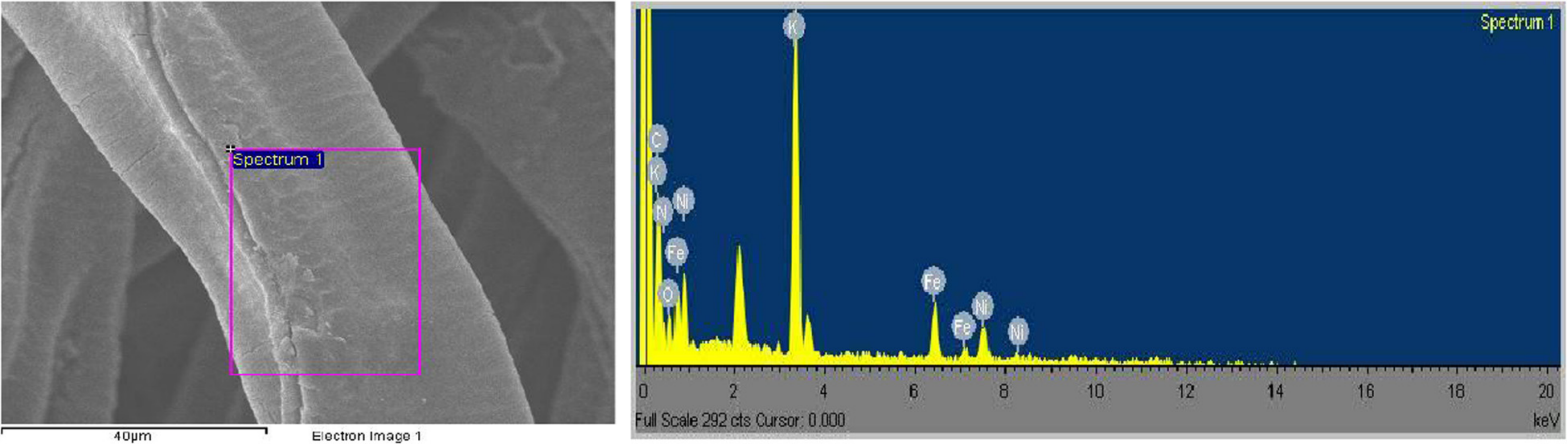
EDS spectrum of the surface part of the KNiHCF-loaded PP fiber.

Figure 2 shows the EDS spectrum of the outer surface of the KNiHCF-loaded PP fiber. The peaks corresponding to C, N, O, K, Fe, and Ni in the EDS spectrum confirm the presence of KNiHCF phase in the synthesized nanocomposite fabric. According to the results presented in Table 1, the chemical formula of KNiHCF is close to K_2_Ni[Fe(CN)_6_].

**Table 1:** **Results of the EDS analysis of the outer surface of the KNiHCF**-**loaded PP fabric**

**Element**	**Weight percent**	**Atomic percent**
C K	34.23	46.01
N K	28.90	33.31
O K	12.10	12.22
K K	11.29	4.66
Fe K	6.60	1.91
Ni K	6.88	1.89
Total	100.00	

**Figure 3:**
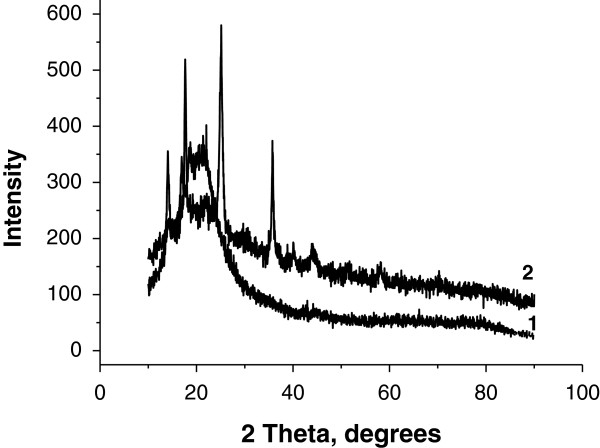
X-ray diffractograms of the original PP fabric (1) and synthesized nanocomposite KNiHCF-loaded PP fabric (2).

The X-ray diffractograms of the original PP fabric (1) and the synthesized KNiHCF-loaded PP fabric (2) are depicted in Figure 3. The well-defined peaks on the nanocomposite's diffractogram indicate the crystalline structure of the KNiHCF nanoparticles. Main diffraction peaks at 2*θ* values of 17.5°, 25.1°, 30.6°, 35.6°, 40.4°, and 44.5° are attributed to the Miller indexes of (200), (220), (222), (400), (420), and (422) of the diffraction planes, respectively, indicating the crystalline face-centered cubic structure of the KNiHCF nanoparticles, which match well with those reported for K_2_Ni[Fe(CN)_6_] (JCPDS Card No. 20-0915). The calculated lattice parameter *a* is 10.06 ± 0.04 Å, and it is agreed well with those reported previously [9].

**Figure 4:**
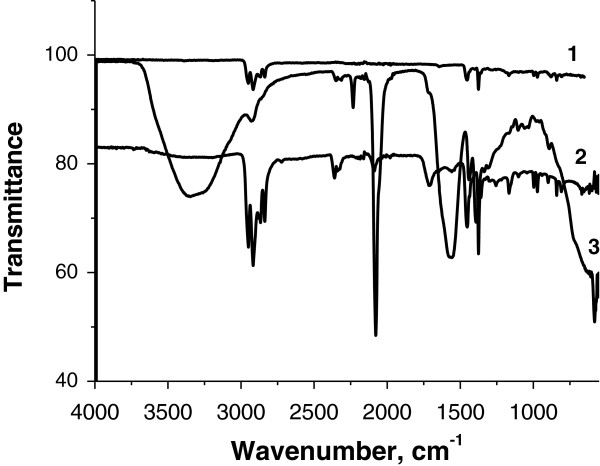
FT-IR-ATR spectra of PP (1), PP-g-PAA (2), and KNiHCF-loaded PP fabrics (3).

Figure 4 shows the FT-IR-ATR spectra of the PP (1), PP-g-PAA (2), and KNiHCF-loaded PP fabrics (3). The sharp and strong absorption peak in spectrum 3 at 2,090 cm^−1^ corresponds to the stretching vibration of the C ≡ N group. Furthermore, the weak peaks (3,420 and 3,265 cm^−1^) in the broad region of 3,000 to 3,650 cm^−1^ are related to the stretching vibration of interstitial water.

### Cesium adsorption studies

The adsorption of cesium ions on potassium nickel hexacyanoferrate proceeds via stoichiometric ion exchange between the potassium and cesium ions. To investigate the efficiency of the synthesized nanocomposite KNiHCF-loaded PP fabric, the effect of contact time, pH, and sodium ion concentration on cesium ion adsorption was investigated in detail.

#### Effect of contact time on cesium ion adsorption

**Figure 5:**
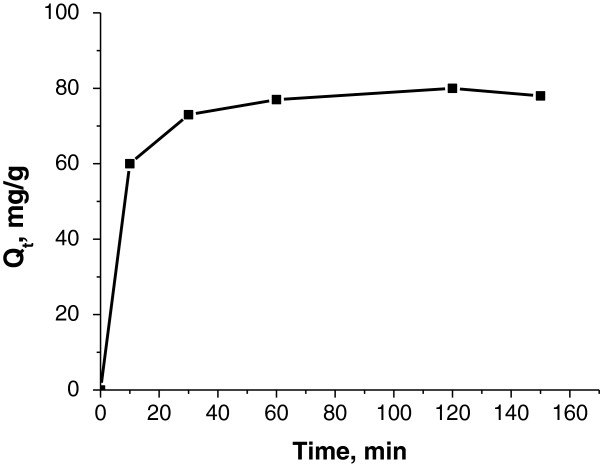
**Effect of contact time on the amount of Cs ions adsorbed by the KNiHCF-loaded PP fabric.** Initial cesium concentration = 780 mg/l; pH ~ 9.

Figure 5 shows the effect of contact time on the amount of Cs ions adsorbed by the synthesized nanocomposite adsorbent. It can be seen that cesium adsorption is a rapid process; the major fraction (>95%) of the cesium ions presented in the solution was adsorbed within the first 30 min. The equilibrium amount of Cs adsorbed is 78 mg/g.

#### Effect of pH on cesium ion adsorption

**Figure 6:**
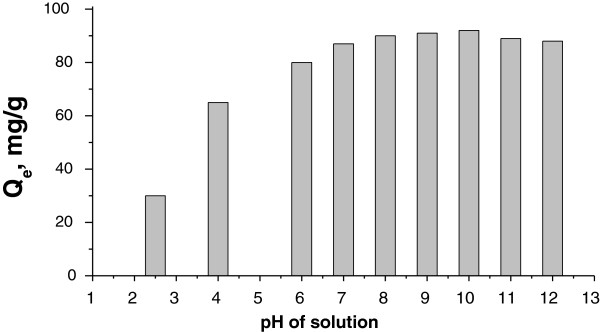
**Effect of pH on the adsorption of cesium ions onto the KNiHCF-loaded PP fabric.** Initial cesium concentration = 1,000 mg/l.

The effect of pH on the equilibrium amount of Cs ions adsorbed was studied by varying the pH of the initial solution. It can be seen (Figure 6) that the *Q*_*e*_ value does not change much in the pH range from 6 to 12. These results suggest that the synthesized adsorbent can be effectively used for adsorption of cesium ions over a wide pH range, but more effectively in neutral and basic solutions.

#### Effect of sodium ion concentration on cesium ion adsorption

**Figure 7:**
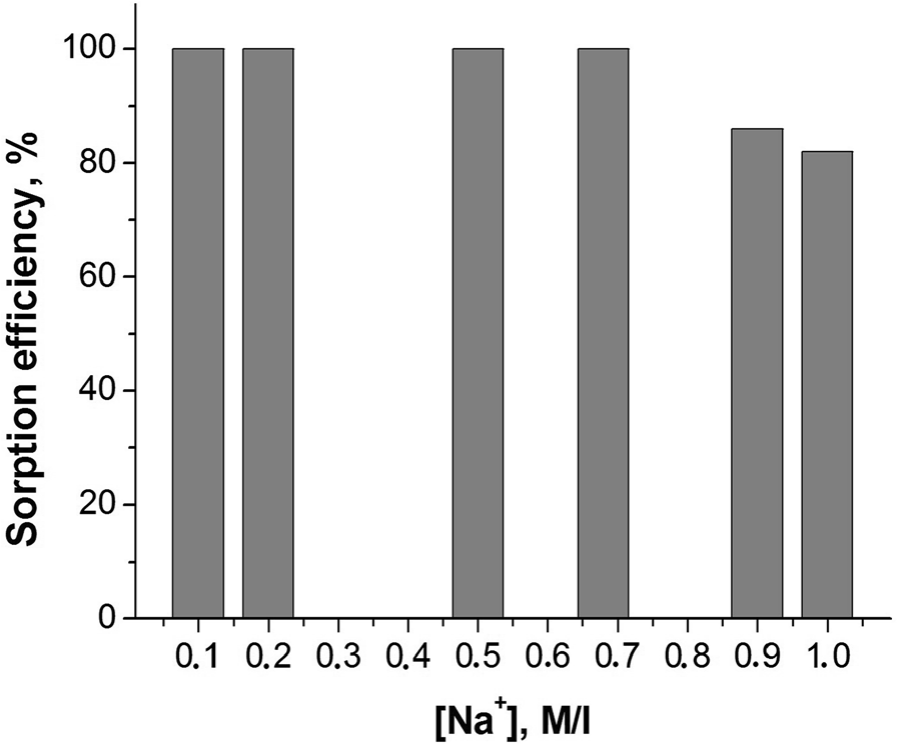
**Effect of sodium ion concentration on the adsorption efficiency of the KNiHCF-loaded PP fabric.** Initial cesium concentration = 36 mg/l; pH ~ 9.

The adsorption of cesium ions depends on the concentration of competitive ions. In this study we considered the competition of sodium ions with respect to the adsorption of cesium ions. Sodium ions are abundant in both seawater and freshwater, and they are the main chemical constituent in a typical evaporator concentrate from nuclear power plants [3]. The effect of competitive sodium ions on the adsorption efficiency of the KNiHCF-loaded PP fabric was studied keeping the concentration of cesium ions constant (36 mg/l or 0.026 mM/l) and varying the concentration of sodium ions (0.1 to 1 M/l) under basic condition (pH ~ 9.0). Figure 7 indicates that within the sodium concentration range of 0.1 to 0.68 M/l (where the ratio Na/Cs ≤2,615), the cesium adsorption efficiency has a maximum and decreases with the increase in sodium ion concentration up to the studied concentration of 1.0 M/l. These results indicate that the adsorption efficiency of cesium ions is affected by the presence of sodium ions in the solution due to the competition of sodium ions for available exchange sites. However, the observed results testify to the high selectivity of the synthesized composite adsorbent to cesium ions, and it can be used efficiently even in the presence of high concentrations of sodium ions. It should be noted that typical divalent cations such as Ca, Mg, Cu, and Pb show no or very little effect on cesium ion adsorption efficiency by HCFs.

## Conclusions

A novel composite adsorbent based on polypropylene fabric with chemically bound nanoparticles of potassium nickel hexacyanoferrate was successfully prepared by a two-stage experiment: radiation-induced graft polymerization of acrylic acid onto the surface of nonwoven polypropylene fabric followed by the *in situ* formation of KNiHCF nanoparticles and their stabilization on the fabric surface within the grafted chains.

SEM, FT-IR-ATR, and X-ray diffraction techniques confirmed the formation of KNiHCF as crystalline nanoparticles with a face-centered cubic structure. The cesium adsorption on the composite adsorbent based on the KNiHCF-loaded PP fabric was studied as a function of contact time, pH, and the presence of competitive sodium ions. The synthesized adsorbent demonstrated fast adsorption kinetics, high adsorption capacity over a wide pH range, and selectivity in removal of Cs ions from an alkaline solution with a high concentration of competitive sodium ions.

## Competing interests

The authors declare that they have no competing interests.

## Authors’ contributions

YB synthesized the KNiHCF-loaded polypropylene fabric, wrote the manuscript, plotted the graphs, submitted the manuscript to the journal, and revised it. SK carried out the Cs analyses in the studied solutions. DHH carried out the radiation-induced graft polymerization experiment. HKC performed the FT-IR-ATR and SEM investigations. All authors read and approved the final manuscript.
